# New approaches to pharmacosurveillance for monitoring prescription frequency, diversity, and co-prescription in a large sentinel network of companion animal veterinary practices in the United Kingdom, 2014–2016

**DOI:** 10.1016/j.prevetmed.2018.09.004

**Published:** 2018-11-01

**Authors:** D.A Singleton, F. Sánchez-Vizcaíno, E. Arsevska, S. Dawson, P.H. Jones, P.J.M. Noble, G.L. Pinchbeck, N.J. Williams, A.D. Radford

**Affiliations:** a Institute of Infection and Global Health, University of Liverpool, Leahurst Campus, Chester High Road, Neston, CH64 7TE, United Kingdom; b National Institute for Health Research, Health Protection Research Unit in Emerging and Zoonotic Infections, The Farr Institute @ HeRC, University of Liverpool, Waterhouse Building, Liverpool, L69 3GL, United Kingdom; c Institute of Veterinary Science, University of Liverpool, Leahurst Campus, Chester High Road, Neston, CH64 7TE, United Kingdom

**Keywords:** Companion animal, Co-prescription, Pharmacosurveillance, Prescription, Small animal, Surveillance

## Abstract

Pharmaceutical agents (PAs) are commonly prescribed in companion animal practice in the United Kingdom. However, little is known about PA prescription on a population-level, particularly with respect to PAs authorised for human use alone prescribed via the veterinary cascade; this raises important questions regarding the efficacy and safety of PAs prescribed to companion animals. This study explored new approaches for describing PA prescription, diversity and co-prescription in dogs, cats and rabbits utilising electronic health records (EHRs) from a sentinel network of 457 companion animal-treating veterinary sites throughout the UK over a 2-year period (2014–2016).

A novel text mining-based identification and classification methodology was utilised to semi-automatically map practitioner-defined product descriptions recorded in 918,333 EHRs from 413,870 dogs encompassing 1,242,270 prescriptions; 352,730 EHRs from 200,541 cats encompassing 491,554 prescriptions, and 22,526 EHRS from 13,398 rabbits encompassing 18,490 prescriptions respectively. PA prescription as a percentage of booked consultations was 65.4% (95% confidence interval, CI, 64.6–66.3) in dogs; in cats it was 69.1% (95% CI, 67.9–70.2) and in rabbits, 56.3% (95% CI, 54.7–57.8). Vaccines were the most commonly prescribed PAs in all three species, with antibiotics, antimycotics, and parasiticides also commonly prescribed. PA prescription utilising products authorised for human use only (hence, ‘human-authorised’) comprised 5.1% (95% CI, 4.7–5.5) of total canine prescription events; in cats it was 2.8% (95% CI, 2.6–3.0), and in rabbits, 7.8% (95% CI, 6.5–9.0). The most commonly prescribed human-authorised PA in dogs was metronidazole (antibiotic); in cats and rabbits it was ranitidine (H_2_ histamine receptor antagonist). Using a new approach utilising the Simpson’s Diversity Index (an ecological measure of relative animal, plant etc. species abundance), we identified differences in prescription based on presenting complaint and species, with rabbits generally exposed to a less diverse range of PAs than dogs or cats, potentially reflecting the paucity of authorised PAs for use in rabbits. Finally, through a novel application of network analysis, we demonstrated the existence of three major co-prescription groups (preventive health; treatment of disease, and euthanasia); a trend commonly observed in practice.

This study represents the first time PA prescription has been described across all pharmaceutical families in a large population of companion animals, encompassing PAs authorised for both veterinary and human-only use. These data form a baseline against which future studies could be compared, and provides some useful tools for understanding PA comparative efficacy and risks when prescribed in the varied setting of clinical practice.

## Acronyms

EHR: Electronic health record
PA: Pharmaceutical agent
PC: Pharmaceutical class
PD: Prescription diversity
PF: Pharmaceutical family
SAVSNET: Small animal veterinary surveillance network

## 1 Introduction

Pharmaceutical agent (PA) prescription is an essential component of companion animal veterinary practice. However, lack of available, structured population-level data on PA prescription has resulted in a limited capacity to assess the comparative efficacy and risks of PAs being prescribed to clinically-affected animals living outside of the controlled conditions of a clinical efficacy trial (pharmacosurveillance). The increasing digitisation of animal health records, most notably in developed countries, together with recent advances in health informatics research, has provided an unprecedented opportunity to fill this gap (O’Neill et al., 2014; Sánchez-Vizcaíno et al., 2015).

In human medicine, the utility of electronic health records (EHRs) for effective pharmacosurveillance has been well demonstrated. Such systems have the potential to provide rapid monitoring and communication of population experience (Eguale et al., 2010; Rowlingson et al., 2013), identifying rare events as well as previously unseen variability in response to PA prescription associated with sub-population genetic diversity (Patel and Kaelber, 2014; Tamblyn et al., 2016). Key areas of pharmacosurveillance research have focused on adverse drug reactions and polypharmacy, the latter being defined as concurrent prescription of multiple distinct PAs to a single person or animal (Eguale et al., 2010; Cavallo et al., 2013; Sutherland et al., 2015; Urfer et al., 2016).

Expanding prescription surveillance beyond adverse drug reaction monitoring (pharmacovigilance, the ‘risks’) to also include PA efficacy assessment (pharmacosurveillance, the ‘benefits and the risks’) would represent a significant development for companion animal veterinary practice. In the United Kingdom, pharmacovigilance is led by the Veterinary Medicine Directorate through a voluntary reporting service (Veterinary Medicines Directorate, 2017). Whilst such services provide population-level data on adverse drug reactions, they are generally associated with an un-quantified level of under-reporting. When a PA is not available to treat a particular condition in a species under their care, the veterinary surgeon responsible for the animal may in particular circumstances (for example to avoid causing unacceptable suffering) prescribe an unauthorised PA under the veterinary cascade; these might include PAs authorised for use in other animal species, PAs authorised for human use only (hence, ‘human-authorised’), or imported medicines (Veterinary Medicines Directorate, 2013; Ramsey, 2017a, Ramsey, 2017b). Currently there are no means by which human-authorised PA prescription can be monitored at the population-level; this raises important questions regarding the efficacy and safety of such PAs when prescribed to companion animals.

More recently, as EHRs have become more available, they have been used to address questions of timely importance, particularly relating to antibiotic prescription in light of the global concern relating to antimicrobial resistance (Mateus et al., 2011; Radford et al., 2011; Buckland et al., 2016; Singleton et al., 2017). Although these studies have shown important trends in antibiotic prescription, to date they have not been able to look at concurrent use of other PAs within the same consultation (co-prescription), despite evidence of antibiotics being co-prescribed with other non-antibiotic PAs (German et al., 2010; Jones et al., 2014). Developing an understanding of what veterinary surgeons prescribe in addition to, or instead of, antibiotics will be necessary to enhance stewardship at the population-level.

Whilst these studies point to the potential of veterinary EHRs to augment existing pharmacosurveillance, two features must be addressed before the full benefits of these new data can be realised. Firstly, the scale and complexity of EHR databases require new ways to analyse health records, and generate population-level statistics. Secondly, due to a lack of a centrally agreed PA classification and terminology system within the veterinary sector O’Neill et al., 2014), individual veterinary practices frequently develop their own personalised nomenclatures when referring to for PA prescription within the EHR. In this regard, categorising PAs to an agreed and common ontology may be more complex in animal rather than human health, the latter frequently benefitting from the use of standardised PA lists (Tamblyn et al., 2016).

In this study, we have addressed the issues of prescription data scale, complexity and inconsistency using data collected over 2 years from a large sentinel network of UK-based companion animal veterinary practices. We describe a text mining approach to map variable practitioner-defined PAs to taxonomies, and propose two new metrics to summarise population-level PA prescription, namely prescription diversity and social network analysis for co-prescription visualisation. Using these combined approaches, we have been able to describe, in various degrees of detail, intra- and inter-species variability in the manner with which PAs are prescribed in companion animal practice.

## 2 Materials and methods

### 2.1 Data collection

Electronic Health Records (EHRs) were collected in near real-time from booked consultations in volunteer UK veterinary practices taking part in the Small Animal Veterinary Surveillance Network (SAVSNET) between 1 April 2014 and 31 March 2016. A more complete description of the data collection protocol has been previously described (Sánchez-Vizcaíno et al., 2015). A veterinary practice (*n* = 216) was defined as a single veterinary business, whereas a site (s) (*n* = 457) also included all branches that form a veterinary practice. In addition to a range of animal signalment data (e.g. age, sex, breed etc.), each EHR also included a free text product description defined by individual practices to record everything supplied at the time of each consultation. At the end of each consultation, practitioners participating in SAVSNET also code each EHR by selecting one of ten main presenting complaints consisting of gastroenteric, respiratory, pruritus, trauma, tumour, kidney disease, other unwell, post-operative, vaccination and other healthy to indicate the main reason why the animal presented for the given consultation (Supplementary Material, Table 1).

### 2.2 Pharmaceutical agent prescription identification

The product description field of the EHR was utilised to identify Pharmaceutical Agent (PA) prescription. Initially, a set of 52,267 product descriptions (1 April 2014 - 26 August 2015) were manually determined to contain PA prescription by reference to the Veterinary Medicine Directorate’s Product Information Database for veterinary authorised PAs, and the electronic Medicines Compendium (Datapharm Communications) for PAs authorised for human use alone. PAs were manually categorised, via partial reference to the ATCvet reference schema (World Health Organisation, 2018), into a hierarchical structure whereby each PA could be summarised into both a pharmaceutical family (PF) e.g. antibiotic, and pharmaceutical class (PC) e.g. fluoroquinolone. A total of 26 PFs and 274 PCs were identified. The classification system used can be downloaded as a supplement to this article. PAs were further categorised into those authorised for veterinary use and those authorised for human use only. A third group was devised (hence, ‘generic’) that consisted of active substances authorised for prescription in both humans and animals, but was recorded within the EHR using a term which did expressly state whether the product was veterinary- or human-authorised e.g. ‘co-amox’. Whilst the majority of product descriptions could be identified to specific active substance, the ‘vaccine’, ‘neurological’, ‘euthanasia’ and ‘replacement agent’ (veterinary- or human-authorised pharmaceutical products intended for rehydration, vitamin and/or mineral replacement) PFs frequently contained descriptors where we were unable to determine the specific PA prescribed e.g. ‘1x dog annual booster’. Hence, it was not possible to summarise these PFs beyond family level.

Following this initial manual categorisation, a list of prescription-identifying strings (*n* = 1984) was formulated to identify prescription-containing product descriptions in a new larger data set suitable for further analysis (*n* unique product descriptions = 95,709; 1 April 2014 - 31 March 2016). Before these strings were applied to the dataset a sequence of regular expressions were applied to product descriptions to exclude those relating to diagnostic tests which could be misidentified as containing a PA prescription e.g. phenobarbitone toxicity test (5318 unique product descriptions excluded); insulin syringes which could be mistaken for a prescription of insulin (211 unique product descriptions excluded), and product descriptions containing references to a refund (22 unique product descriptions excluded) (Supplementary Material, Table 2). PA prescription identifying strings were then applied, with a total of 1759 strings being utilised to identify 56,699 unique product descriptions pertaining to a PA prescription.

### 2.3 Pharmaceutical agent prescription diversity

We define PA Prescription Diversity (PD) as ‘the frequency and variety with which a practice prescribes pharmaceutical classes (PC) within a determined pharmaceutical family (PF)’. In this study, we calculate PD using an adaptation of the Simpson’s Diversity Index, an equation utilised most often in ecology to provide a quantitative measure of relative animal, plant etc. species abundance within a particular geographical area over a defined timeframe (Allaby, 2010), adjusted to a 0–1 scale where 1 represents maximal diversity. We intend this measure to distil otherwise complex prescription data into a single metric that summarises the propensity of prescribers towards particular PCs within a PF. The PD equation is as follows:

$${PD}_{i}=1-\;\frac{\sum {np}_{i}({np}_{i}-1)}{{NP}_{i}({NP}_{i}-1)}$$

Where *i* = individual practice; *np* = number of prescriptions of a particular PC within a PF, and *NP* = total number of prescriptions within a PF. For example, take practice ‘A’: a particular PF, ‘X’, contains 4 PCs known as X1, X2, X3 and X4 respectively. Practice ‘A’ prescribed a PA within the PF ‘X’ on 2000 occasions within the surveillance period, these PA prescriptions were split by PC as follows: X1 = 800, X2 = 400, X3 = 400, X4 = 400. The completed equation would therefore appear as thus:

PDA=1-((800×800-1+(400×400-1+(400×400-1 +400×400-12000×(2000-1)

*PD_A_* = 0.72

Due to some PFs containing a greater range of available PCs, each PF will possess a natural PD limit. For example, a ‘completely diverse’ PF (where all available PCs have been prescribed evenly) containing four PCs will possess a PD of 0.75, whereas a PF containing eight PCs will possess a PD of 0.88. As such, PD should be interpreted only within a PF, and should not be used for comparisons with other PFs. Here, we benchmarked practices against each other, rather than against each PFs theoretical maximum PD, to avoid establishing practically unreachable ‘targets’.

For brevity we demonstrated the application of the PD metric using the five main PFs associated with treating infectious agents (antibiotic, antimycotic, ectoparasiticide, endectocide and endoparasiticide); these are all commonly prescribed in all three species and have critical relevance in the context of antimicrobial resistance to both animals and humans (Cuny et al., 2015; Zhang, 2016).

### 2.4 Co-prescription network

**Fig. 1:**
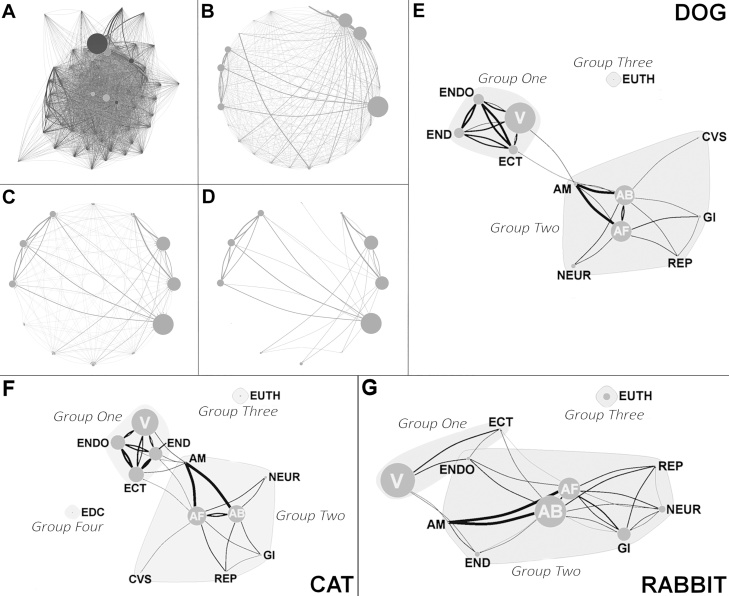
Pharmaceutical co-prescription network model construction, sparsification and formation to detect group structure. Size of each Pharmaceutical Family (PF) node is relative to number of prescriptions, and edge width is relative to frequency of co-prescription between each PF. Diagrams refer to points along final model formation as follows: (A) Pharmaceutical class-level co-prescription network; (B) PF-level co-prescription network; (C) Prescription frequency sparsified; (D) Co-prescription frequency sparsified; (E) Dogs: Final model with group structure (E), cats (F), and rabbits (G). AB = antibiotic; AF = anti-inflammatory; AM = antimycotic; CVS = cardiovascular; ECT = ectoparasiticide; EDC = endocrine; END = endectocide; ENDO = endoparasiticide; EUTH = euthanasia; GI = gastrointestinal; NEUR = neurological; REP = replacement agent; V = vaccine.

Co-prescription was here defined as prescription of two or more PAs belonging to a different PF within the same booked consultation. Three co-prescription networks were created for each species, using social network analytical methods, via the igraph package available in R (Csardi, 2006). A node referred to a particular PF, with the size of the node being relative to frequency of prescription. An edge referred to a co-prescription event between different PFs, with edge weight being relative to frequency of co-prescription. As a number of PFs were rarely prescribed and some co-prescription links were uncommon, the networks were then sparsified, retaining PF nodes that contributed 0.5% or more to total prescriptions, and edges that contributed in excess of the mean co-prescription frequency for each of the three networks. The network was then examined for group structure via a propagating label algorithm, with the aim of defining PF groups of preferential co-prescription. In short, this algorithm utilised the structure of the network itself by enabling a uniquely labelled node to adopt the label that the majority of its neighbours possess in an iterative process until groups of densely connected nodes (if present) formed a consensus on a single label, indicating presence of a group (Raghavan et al., 2007). A visual summary of this methodological process can be observed in Fig. 1.

### 2.5 Statistical analysis

Consultation and prescription-level proportions and confidence intervals were calculated to adjust for clustering (bootstrap method, *n* = 5000 samples) within sites and at animal level within practices (Lesnoff and Lancelot, 2012). Repeat prescriptions for the same animal were considered separately in consultation-level analyses. A pairwise Wilcoxon rank sum test with Bonferroni correction to account for multiple comparisons (hence, ‘Wilcoxon’) was performed to examine PF variability (calculated at practice-level) between animal species for selected PFs. Kendall correlations (*t* test to reject null hypothesis) were performed for testing the association between PD and the total number of consults contributed by each practice, and PD and the number of PA prescription events by each practice for dogs, cats, and rabbits for selected PFs. Statistical significance was defined as *P* <  0.05 and all analyses were carried out using R version 3.4.1 (R Core Team, 2017).

## 3 Results

Data were available from 413,870 dogs (from 918,333 Electronic Health Records, EHRs), 200,541 cats (352,730 EHRs) and 13,398 rabbits (22,526 EHRs). This yielded a total number of 1,241,888 recorded Pharmaceutical Agent (PA) prescription events in dogs; 490,872 in cats, and 18,530 in rabbits. The percentage of booked consultations where at least one prescription was recorded was 65% (95% confidence interval (CI) 65-66) in dogs; whereas in cats it was 69% (95% CI 68-70), and in rabbits it was 56% (95% CI 55-58). Dogs recorded at least one prescription event for all 26 Pharmaceutical Families (PFs) whereas cats recorded prescription for 24 PFs and rabbits, 17 PFs. Vaccines were the most commonly prescribed PF in all species, though rabbits (22%, 95% CI 20-23) were less commonly prescribed vaccines than dogs (28%, 95% CI 27-29) or cats (30%, 95% CI 29-31). Conversely, rabbits were euthanised the most frequently (4%, 95% CI 4-5) compared to dogs (1%, 95% CI 1-1) or cats (2%, 95% CI 2-2) (Table 1). At the individual animal level, 81% of dogs (95% CI 80–82) and 81% of cats (95% CI 80-82) had been prescribed at least one PA within the 2-year study period; levels were lower in rabbits (68%; 95% CI 67-70).

**Table 1:** Rate per 10,000 consultations for dogs, cats and rabbits where a particular pharmaceutical family was prescribed. A blank space signifies no prescription of the PF in that species.

Pharmaceutical family	Dog		Cat		Rabbit	
	Rate per 10,000 consults	95% Conf. interv.	Rate per 10,000 consults	95% Conf. interv.	Rate per 10,000 consults	95% Conf. interv.
Allergy	36	31–42	5	4–6		
Anabolic	2	2–3	34	25–43	1	0-3
Anti-infective	0.01	0.00–0.03				
Anti-inflammatory	1916	1848–1982	1783	1711–1857	1246	1170–1322
Antibiotic	1879	1820–1938	1749	1685–1814	1653	1551–1754
Antimycotic	394	380–408	74	69–79	52	41–63
Antiviral	0.01	0.00–0.03	2	1-2		
Bladder	0.03	0.00–0.08	0.3	0.0-0.5		
Cardiovascular	101	94–108	137	124–151	11	6–16
Chemotherapeutic	3	2–4	3	2-4		
Diagnostic	0.2	0.1–0.4	0.1	0.0–0.2	0.4	0.0–1.3
Ectoparasiticide	965	882–1048	1342	1248–1435	124	103–145
Endectocide	1161	1069–1252	1673	1570-1775	334	300–369
Endocrine	43	38–48	142	129–155		
Endoparasiticide	944	872–1016	1415	1332–1500	246	209–282
Euthanasia	103	97–110	229	217–240	431	395–467
Gastrointestinal	263	247–278	196	178–214	624	565–683
Hormone	37	33–40	14	8–20	2	0–4
Immunosuppression	34	31–38	6	5–7		
Liver	3	3–4	4	3–4		
Neurological	378	348–407	214	190–238	263	209–317
Ocular	19	14–25	5	4-6	12	5–18
Renal	50	20–70				
Replacement agent	49	42–56	129	111–146	74	52–96
Respiratory	13	11–16	28	23–32	12	7–18
Vaccine	2792	2700–2885	3023	2913–3132	2159	2020–2298

### 3.1 Authorisation

Overall prescription of PAs authorised for human use only (human-authorised) were relatively low in each species. In dogs, 5% (95% CI 5-5) were human-authorised, 93% (95% CI 93-94) of prescriptions were authorised for veterinary use, and 2% (95% CI 2-2) generic. In cats 3% (95% CI 3-3) were human-authorised, 96% (95% CI 96-96) veterinary-authorised, and 1% (95% CI 1-2) generic. In rabbits 8% (95% CI 7-9) were human-authorised, 90% (95% CI 88-91) veterinary-authorised, and 2% (95% CI 2-3) generic.

A summary of the percentage of PA prescriptions which utilised products authorised for human-only use by PF can be seen in Table 2. The three most commonly prescribed human-authorised PAs in dogs were metronidazole (antibiotic), tramadol (neurological), and ranitidine (gastrointestinal), representing 16%, 12% and 12% of total human-authorised prescriptions in dogs, respectively. This compared with ranitidine (gastrointestinal), dexamethasone (anti-inflammatory) and neomycin sulphate (antibiotic) in cats, representing 13%, 8% and 7% of total human-authorised prescriptions in cats, respectively. The three most commonly prescribed human-authorised PAs in rabbits were ranitidine (gastrointestinal), trimethoprim potentiated sulfamethoxazole (antibiotic) and cisapride (gastrointestinal), representing 38%, 10% and 9% of total human-authorised prescriptions in rabbits, respectively.

**Table 2:** Summary of pharmaceutical agent prescriptions for dogs, cats, and rabbits summarised by pharmaceutical family, and the percentage of total prescription events for each PF that included products authorised for human use only (Human).

Pharmaceutical family	Dog		Cat		Rabbit	
	Number (%) of prescription events	Human (%)	Number (%) of prescription events	Human (%)	Number (%) of prescription events	Human (%)
Vaccine	324727 (26.1)	–a	110245 (22.5)	–	4907 (26.5)	–
Antibiotic	218700 (17.6)	11.4	71088 (14.5)	5.7	4481 (24.2)	10.9
Anti-inflammatory	209644 (16.9)	3.2	77244 (15.7)	1.6	3132 (16.9)	3.6
Endectocide	113887 (9.2)	0.0	63401 (12.9)	0.0	765 (4.1)	0.0
Endoparasiticide	108802 (8.8)	0.0	59997 (12.2)	0.0	556 (3.0)	0.0
Ectoparasiticide	98768 (8.0)	0.0	55895 (11.4)	0.0	285 (1.5)	0.0
Neurological	49241 (4.0)	–	11,899 (2.4)	–	1037 (5.6)	–
Antimycotic	36766 (3.0)	0.3	2611 (0.5)	0.1	118 (0.6)	0.8
Gastrointestinal	30981 (2.5)	33.9	8962 (1.8)	51.3	1914 (10.3)	35.8
Cardiovascular	14554 (1.2)	5.2	6720 (1.4)	26.8	29 (0.2)	34.5
Euthanasia	10057 (0.8)	–	8458 (1.7)	–	1051 (5.7)	–
Replacement agent	7320 (0.6)	–	5316 (1.1)	–	188 (1.0)	–
Endocrine	4235 (0.3)	14.8	5443 (1.1)	0.7	0	
Hormone	3452 (0.3)	0.3	496 (0.1)	0.0	5 (0.03)	0.0
Allergy	3381 (0.3)	100.0	186 (0.04)	100.0	0	
Immunosuppression	3285 (0.3)	7.1	207 (0.04)	1.0	0	
Ocular	1861 (0.1)	99.9	186 (0.04)	100.0	27 (0.2)	100.0
Respiratory	1305 (0.1)	2.5	1010 (0.2)	6.8	31 (0.2)	0.0
Chemotherapeutic	319 (0.03)	87.1	116 (0.02)	94.0	0	
Liver	304 (0.02)	100.0	125 (0.03)	100.0	0	
Anabolic	219 (0.02)	0.0	1201 (0.2)	0.0	3 (0.02)	0.0
Renal	49 (0.004)	100.0	0		0	
Diagnostic	26 (0.002)	100.0	4 (0.001)	100.0	1 (0.01)	100.0
Bladder	3 (0.0002)	100.0	9 (0.002)	100.0	0	
Anti-infective	1 (0.0001)	100.0	0		0	
Antiviral	1 (0.0001)	0.0	53 (0.01)	66.0	0	

a Not able to accurately estimate.

### 3.2 Pharmaceutical class and prescription diversity

For the five PFs analysed (antibiotic, antimycotic, ectoparasiticide, endectocide and endoparasiticide) we found key differences between species at the level of PC, particularly for antibiotic prescription. For dogs, the most commonly prescribed antibiotic was clavulanic acid potentiated amoxicillin (29% of antibiotic prescriptions); for antimycotics, imidazoles (60%); for ectoparasiticides, neonicotinoids (76%); for endectocides, milbemycin (97%), and for endoparasiticides, quinolines (78%) (Supplementary Material, Table 3).

For cats, the most commonly prescribed antibiotic was 3^rd^ generation cephalosporins (36% of antibiotic prescriptions); for antimycotics, polyenes (58%); for ectoparasiticides, neonicotinoids (65%); for endectocides, milbemycin (87%), and for endoparasiticides, quinolines (81%) (Supplementary Material, Table 4).

For rabbits, fluoroquinolones represented the most commonly prescribed antibiotic (49% of antibiotic prescriptions); for antimycotics, polyenes (52%); for ectoparasiticides, insect growth regulators (59%); for endectocides, avermectins (98%), and for endoparasticides, benzimidazoles (98%) (Supplementary Material, Table 5).

PD was calculated for each of the five exemplar PFs across all contributing veterinary practices (Table 3). Dogs (median PD 0.83) showed significantly greater antibiotic PD compared to cats (median PD 0.75) and rabbits (median PD 0.64). Similarly, cats reported greater PD compared to rabbits (Wilcoxon *P* <  0.001 for all comparisons). In dogs, practices reporting higher PD values tended to contribute more canine consultations (Kendall correlation, τ = 0.18, *P* <  0.001) and more antibiotic prescriptions (Kendall correlation, τ = 0.17, *P* <  0.001) to the project, though these relationships were weak.

**Table 3:** Summary of pharmaceutical classes prescribed and median prescription diversity (PD) for five exemplar pharmaceutical families in dogs, cats and rabbits.

Pharmaceutical family	Animal species	Total classes prescribed	Median classes prescribed	Median PD
Antibiotic	Dog	21	13	0.83
	Cat	20	10	0.75
	Rabbit	17	4	0.64
Antimycotic	Dog	8	2	0.44
	Cat	4	2	0.44
	Rabbit	4	1	0.00
Ectoparasiticide	Dog	9	5	0.14
	Cat	7	3	0.23
	Rabbit	5	2	0.33
Endectocide	Dog	2	2	0.00
	Cat	2	2	0.02
	Rabbit	2	1	0.00
Endoparasiticide	Dog	4	3	0.12
	Cat	4	3	0.14
	Rabbit	4	1	0.00

Considering antimycotic prescription, no significant variation was observed between dogs (median PD 0.44) and cats (median PD 0.44; Wilcoxon *P* =  1.00); dogs and rabbits (median PD 0.00; Wilcoxon *P* =  0.35), or cats and rabbits (Wilcoxon *P* =  0.77). In dogs, practices reporting higher PD values tended to also contribute more consultations (Kendall correlation, τ = 0.13, *P* =  0.01) and antimycotic prescriptions (Kendall correlation, τ = 0.10, *P* =  0.03) to the project, though these relationships were weak.

For ectoparasiticide prescription, no significant variation was observed between dogs (median PD 0.14) and cats (median PD 0.23; Wilcoxon *P* =  0.50); dogs and rabbits (median PD 0.33; Wilcoxon *P* =  1.00), or cats and rabbits (*P* =  1.00). In both dogs (Kendall correlation, τ = 0.25, *P* <  0.001) and cats (Kendall correlation, τ = 0.13, *P* =  0.01), practices reporting higher ectoparasiticide PD values also tended to contribute more consultations to the project, though these relationships were weak.

For endectocide prescription, dogs (median PD 0.00) were significantly less diverse than cats (median PD 0.02) and rabbits (median PD 0.00); cats were significantly more diverse than rabbits (Wilcoxon *P* <  0.001 for all comparisons). In both dogs (Kendall correlation, τ = 0.18, *P* <  0.001) and cats (Kendall correlation, τ = 0.17, *P* <  0.001), practices reporting higher PD values also tended to contribute more consultations, though these relationships were weak.

Regarding endoparasiticide prescription, dogs (median PD 0.12) showed significantly greater diversity compared to rabbits (median PD 0.00; Wilcoxon *P* <  0.001). Cats (median PD 0.14) also showed significantly greater diversity compared to rabbits (Wilcoxon *P* <  0.001), though no significant difference was observed between dogs and cats (Wilcoxon *P* =  1.00). In both dogs and cats, practices reporting higher PD values also tended to contribute a greater number of consultations (Kendall correlation, dogs; τ = 0.16, *P* <  0.001 and cats; τ = 0.25, *P* <  0.001) and prescriptions (Kendall correlation, dogs; τ = 0.10, *P* =  0.03 and cats; τ = 0.22, *P* <  0.001) to the project, though these relationships were weak.

**Fig. 2:**
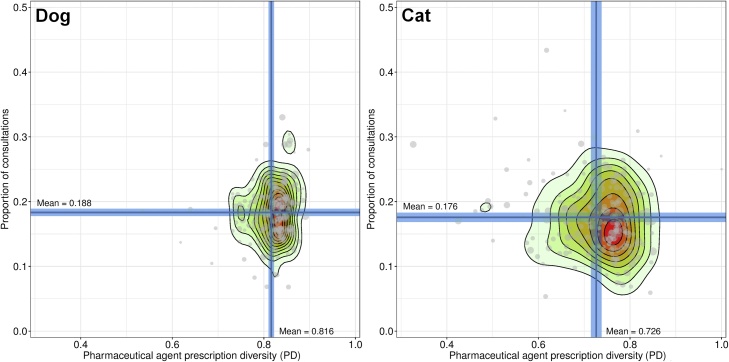
Antibiotic prescription: Practice-level comparison of dog and cat antibiotic prescription diversity (PD) and the proportion of consultations where an antibiotic was prescribed as a proportion of total consultations contributed by each practice (*n* = 216). Blue lines refer to the means and associated 95% confidence intervals of each metric. Point size is relational to number of consultations contributed by each practice where increased point size indicates a greater relative contribution. The contour plot indicates point density, with red indicating maximal point density (For interpretation of the references to colour in this figure legend, the reader is referred to the web version of this article).

**Fig. 3:**
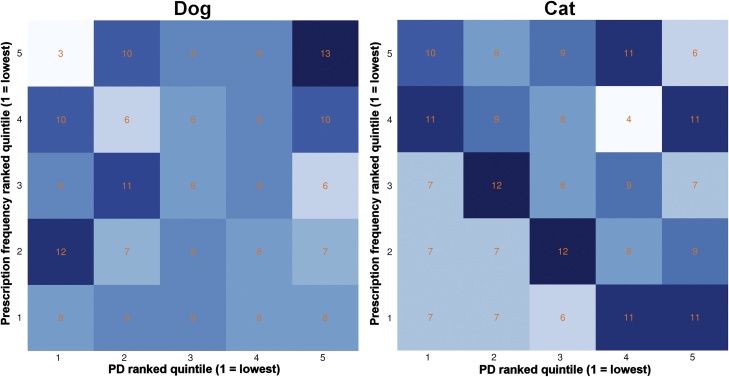
Antibiotic prescription: Dog and cat antibiotic prescription frequency (as a percentage of total consultations) and antibiotic Prescription Diversity (PD) practice-level benchmarking matrix. Prescription frequency and PD were ranked separately by practice (*n* = 216) and sorted into evenly spaced quintiles (1 = lowest prescription frequency and PD) before being summarised by the number of practices that placed into each of the pairwise quintile groups.

For dogs and cats, the antibiotic PF was selected for further demonstration of how PD might be developed into a benchmark; both on a practice and population-level. At the practice-level no significant correlation was observed between antibiotic PD and the frequency with which antibiotics were prescribed as a percentage of total booked consultations (hence, ‘prescription frequency’) in dogs (Kendall correlation, τ = 0.07, *P* =  0.12) or cats (Kendall correlation, τ = −0.07, *P* =  0.11), though a clear single cluster was observed in both species (Fig. 2). Fig. 3 displays a suggested practice summary whereby each contributory practice can be been ranked into quintiles based separately on their values for antibiotic PD and prescription frequency.

Antibiotic prescriptions were finally classified by route of administration and by main presenting complaint. Significant practice-level PD variation was observed between dogs (median PD 0.66) and cats (median PD 0.62) for antibiotics authorised for systemic administration (Wilcoxon *P* <  0.001). Similarly, significant variation was observed between dogs (median PD 0.66) and cats (median PD 0.60) for antibiotics authorised for topical administration (Wilcoxon *P* <  0.001), suggesting that dogs were prescribed a more diverse range of antibiotics than cats across both administration categories. For systemically-authorised antibiotics, PD was further compared against prescription frequency. Some main presenting complaints showed notable variation in PD between species despite similar prescription frequencies e.g. pruritus and post-operative care (Table 4).

**Table 4:** Systemically-authorised antibiotic prescription frequency (percentage of consultations where a systemically-authorised antibiotic was prescribed) and median prescription diversity (PD) in dogs and cats, grouped by main presenting complaint.

Main presenting complaint	Dog		Cat	
	Prescription frequency (%)	Median PD	Prescription frequency (%)	Median PD
Gastroenteric	38.2	0.57	28.9	0.67
Respiratory	40.4	0.56	49.9	0.64
Pruritus	25.5	0.58	24.9	0.38
Trauma	21.3	0.46	50.1	0.56
Tumour	17.5	0.60	19.8	0.60
Kidney disease	26.8	0.45	18.9	0.50
Other unwell	20.3	0.62	24.9	0.61
Post-operative	9.9	0.56	9.6	0.67
Other healthy	1.4	0.66	8.4	0.62
Vaccination	7.0	0.74	1.4	0.67

### 3.3 Pharmaceutical agent co-prescription

In dogs, co-prescription occurred in 40% (*n *= 243,038) of total prescribing consultations; this compared with 45% for cats (*n* = 108,546) and 23% (*n* = 2884) of total prescribing consultations for rabbits. Antibiotics and anti-inflammatories were the most commonly co-prescribed PFs in dogs and rabbits; however in cats the most commonly co-prescribed PFs were endoparasiticides and endectocides (Supplementary Material, Table 6).

Sparsified co-prescription networks for dogs, cats and rabbits fitted to detect group structure can be seen in Fig. 1E–G. Co-prescription formed into three equivalent groups for all three species. The first group (Fig. 3, Group 1) included vaccines and parasiticides; the second group (Fig. 1, Group 2) contained a number of PFs generally focused on treating ‘unhealthy’ animals, with antibiotics and anti-inflammatories being central to this group. The third group (Fig. 1, Group 3) consisted entirely of PAs only associated with euthanasia. Not surprisingly, euthanasia appeared completely separate from any other node in all three networks, suggesting lack of network membership. Cat co-prescriptions formed a fourth isolated group consisting entirely of the endocrine PF (Fig. 1, Group 4).

## 4 Discussion

Electronic health records (EHRs) collected at scale can complement existing pharmacovigilance data, and provide new insight into the efficacy, and risks, of PA prescription and co-prescription in genetically and phenotypically heterogeneous populations. Here we describe a new methodology that enables variable EHR prescription data to be mapped to standardised terms, providing a first broad overview of both veterinary- and human-authorised prescription in a large population of companion animal-treating veterinary practices. We further proposed a new diversity metric for quantifying prescription variability at population- and practice-levels. Finally, co-prescription was common and, through novel application of network analyses, divided into three preferential co-prescription groups corresponding to ‘healthy’, ‘unhealthy’ or ‘euthanasia’ consultations.

Currently, estimates of companion animal veterinary-authorised PA prescription are limited to information provided by Market Authorisation Holders at a wholesale level (Hunt et al., 2015a, Hunt et al., 2015b) and sporadic research papers in response to issues of current importance, such as antimicrobial resistance (Buckland et al., 2016; Singleton et al., 2017). By making PA prescriptions accessible from EHRs, we can now expand monitoring to all PFs across a range of species. We show that PA prescription was common in this population, particularly in dogs and cats in comparison to rabbits. Vaccines were the PF prescribed most frequently in all species, reaffirming the importance of preventive health consultations in veterinary practice (Robinson et al., 2015), and the continued perceived importance of sometimes life threatening vaccine-preventable infections in cats (Day et al., 2016; Afonso et al., 2017), dogs (Clegg et al., 2011; Day et al., 2016) and rabbits (Westcott and Choudhury, 2015).

Population data on PA prescriptions utilising products authorised for human use alone are extremely limited, despite such products forming an important treatment option for companion animals (Buckland et al., 2016; Singleton et al., 2017; Hunt et al., 2015b). Prescription of human-authorised products has the potential to increase the risk of adverse drug reactions in animals, and such cases have been described (Short et al., 2014; Diesel, 2015; Giuffrida, 2016). However, the Market Authorisation Holder has no obligation to investigate adverse drug reaction reports when a human-authorised product is prescribed to an animal (Diesel, 2015), meaning that safety knowledge regarding such products is limited. Here we have been able to identify the most commonly prescribed human-authorised PAs in this population. We found that ranitidine, a H_2_ histamine receptor antagonist, was a commonly prescribed human-authorised PA in all three species, despite another H_2_ histamine receptor antagonist (cimetidine) being authorised for use in dogs. This might be due to a prokinetic effect produced by ranitidine in contrast to cimetidine, or the availability of ranitidine in an oral liquid form, although other factors may be involved (Ramsey, 2017a, Ramsey, 2017b). Although metronidazole was frequently prescribed, the authorisation of a veterinary-authorised metronidazole formulation in December 2015 means that human-authorised metronidazole prescription is likely to decrease (Veterinary Medicines Directorate, 2018). Hunt et al., 2015a, Hunt et al., 2015b previously identified tramadol as a frequently prescribed human-authorised PA in dogs, its popularity possibly due to it being an alternative to non-steroidal anti-inflammatory drugs. We found that rabbits were prescribed human-authorised PAs more frequently than dogs or cats. This might reflect a greater propensity towards prescribing PFs more commonly associated with human-authorised PA prescription e.g. the gastrointestinal PF, and/or a relative paucity of rabbit-authorised PAs - there is little economic benefit to investing in a marketing authorisation for a relatively ‘minor species’.

It is of tantamount importance, especially regarding antibiotic stewardship, to develop summary prescription statistics that are accessible to practising veterinary surgeons, researchers and policy makers alike. In ecology, species diversity calculations are used to summarise species variety and frequency ‘captured’ within a particular area/time (Allaby, 2010). Using similar approaches (modified Peterson Index), interventional studies within human hospitals showed an association between increased antibiotic prescription diversity and concurrent decreased resistant bacteria prevalence (Sandiumenge et al., 2006; Takesue et al., 2010). We initially attempted this approach, though the more varied nature of the SAVSNET population produced findings that were heavily influenced by the number of prescriptions recorded by each veterinary practice (data not presented). It was hence concluded that a modification of the Simpson’s Diversity Index (Allaby, 2010) would provide a more appropriate measure in this population.

In the SAVSNET population we found that rabbits were generally associated with less diverse prescription compared to dogs or cats, possibly again reflecting a paucity of authorised PAs in rabbits. Across all species, in many cases a single PC was pre-dominantly prescribed within a PF, potentially reflecting practice policy and/or PA ‘on-shelf’ availability. We further found variation between clinical presentations not immediately apparent by examining prescription frequency alone. This was particularly apparent for pruritus, where cats displayed reduced systemically-authorised antibiotic prescription diversity compared to dogs, despite similar prescription frequency. This is consistent with the relative prescription dominance of particular antibiotic classes in cats as previously reported (Buckland et al., 2016; Burke et al., 2017; Singleton et al., 2017). A weak, inconsistent level of correlation was noted between prescription diversity and the number of consultations and prescriptions provided by each veterinary practice. This might reflect greater case or care diversity in larger veterinary practices. Of course many factors may impact on prescription diversity in a population such as that sampled by SAVSNET, including market dominance, standard operating procedures, perceived or actual leaders in efficacy, habit and preferred suppliers, especially since the emergence of veterinary sector corporatisation. We propose the methods presented here as a simple means to describe prescription variability between sub-populations at local practice and national levels.

The issue of polypharmacy is of increasing importance to human medicine, particularly in association with an ageing population (Urfer et al., 2016); the same is likely to be true of veterinary medicine (Hunter and Isaza, 2017). Whilst prescription of multiple PAs might be wholly appropriate to combat complex clinical situations, drug-drug interactions have been identified as a major cause of adverse drug reactions (Cavallo et al., 2013; Sutherland et al., 2015). As veterinary prescribing broadens in scope and complexity (Riviere, 2007), there is concern that understanding of veterinary drug-drug interactions is limited (Aidasani et al., 2008), resulting in under-recognition and under-reporting of adverse drug reactions (Hunt et al., 2015a, Hunt et al., 2015b; Belshaw et al., 2016). Instigating routine co-prescription surveillance in veterinary practice would provide clear targets for drug-drug interaction studies that would enable anticipation of, rather than reaction to, adverse drug reaction reports (Aidasani et al., 2008). EHR surveillance could further complement existing voluntary veterinary reporting, potentially also providing real-time feedback of immediate benefit to the patient (Page et al., 2017).

Pharmaceutical co-prescription was shown to be common in all species in this population, though less common for rabbits. Whilst social network analysis has traditionally been associated with social science research, its potential for health research is being increasingly realised (Valente, 2010). There have been some attempts to visualise co-prescription networks in public health studies (Cavallo et al., 2013; Sutherland et al., 2015), though to the authors knowledge, none yet exist in veterinary medicine. The methods presented here identified three similar co-prescription groups summarised as ‘healthy’ consultations primarily concerned with preventive health, ‘unhealthy’ consultations primarily concerned with treating disease, and ‘euthanasia’ consultations. This pattern would be familiar with most veterinary surgeons (Robinson et al., 2015), and provides confidence that this method might be of use for identifying previously unobserved co-prescription trends when applied to particular sub-populations of animals and/or clinical presentations. Cats also demonstrated a fourth group concerned with treatment of endocrine disorders; highlighting increased disorder prevalence in this species e.g. hyperthyroidism (Higgs et al., 2014). Some of the PFs that appeared to be preferentially co-prescribed, notably parasiticides, are likely the result of multivalent products.

There are some limitations with utilising EHRs for the purpose described in this study. Reporting of PA prescriptions depends on veterinary practitioners recording PAs within their practice management software; as such unrecorded products or products described in a fashion that was impossible for the method described here to identify would be missed. Prescription events were considered on a consultation-by-consultation basis; hence, prescription frequency might be relatively over-stated for PAs where repeated prescriptions/applications could be expected, such as vaccinations or PAs associated with management of chronic conditions. Whilst the aim of this study was to broadly monitor and classify PAs into a single hierarchy based on the most likely intended use of such PAs, it is clear that many PAs can be administered for multiple purposes e.g. corticosteroids as anti-inflammatories or immunosuppressives (Cohn, 2010); it was beyond the scope of this study to explore motivation for prescription in individual cases, though this could be achieved via linkage of prescription data presented here to free text information in the clinical narrative (Radford et al., 2011; Burke et al., 2017). Practices contributing data to SAVSNET are recruited on convenience so cannot necessarily be considered to be representative of the wider UK population. The main presenting complaint function does enable all consultations to be encoded; however variable interpretations of the main presenting complaint is possible.

## 5 Conclusions

We have developed pharmacosurveillance tools to enable identification, description and benchmarking of all pharmaceutical agent prescriptions, including those authorised for human use only, utilising EHR data collected from a large-scale population of companion animal-treating veterinary practices. Here we have demonstrated pharmaceutical agents authorised for human use only that are commonly prescribed to companion animals; these should be prioritised for further efficacy and risk investigation. We have established prescription diversity as a useful research tool to identify previously hidden population-level prescription behaviour, and suggest this metric as a useful addition to practice-level benchmarking capability. Finally, though co-prescription data can be of considerable complexity, we have suggested a simple means by which these data can be simplified to uncover findings of value to the veterinary profession.

## Conflict of interest statement

None of the authors of this paper have a financial or personal relationship with other people or organisations that could inappropriately influence or bias the content of this paper.
