# Supplementary material for: New approaches to pharmacosurveillance for monitoring prescription frequency, diversity, and co-prescription in a large sentinel network of companion animal veterinary practices in the United Kingdom, 2014–2016
Source: Prev Vet Med. 2018 Nov 1;159:153–61. doi: 10.1016/j.prevetmed.2018.09.004 (PMC6193134; doi:10.1016/j.prevetmed.2018.09.004)
Supplement: Supplementary file 2 [file mmc2.docx]

**Supplementary Table 1**

Definitions of main presenting complaints as provided to participating veterinary surgeons as a hover box in the United Kingdom Small Animal Veterinary Surveillance Network (SAVSNET) interactive window which appears at the end of each consultation.

| Main presenting complaint | Group | Definition |
| --- | --- | --- |
| Gastroenteric | Unhealthy | Signs including but not limited to diarrhoea, vomiting, weight loss, poor appetite |
| Pruritus | Unhealthy | Signs including but not limited to itching, scratching, pruritic otitis, chewing, licking, rubbing |
| Respiratory | Unhealthy | Signs associated with conditions affecting the upper and / or lower respiratory tract |
| Tumour | Unhealthy | Any suspected or confirmed benign or malignant neoplastic condition |
| Trauma | Unhealthy | Animal suffering a trauma and / or a physical injury |
| Kidney disease | Unhealthy | Signs including but not limited to polydipsia, polyuria, vomiting where kidney disease is a differential |
| Other unwell | Unhealthy | Signs that do not fit in other unwell animal categories including behaviour problems |
| Post-operative | Post-operative | If the animal has presented for post-operative care |
| Vaccination | Healthy | If the animal was booked in for a vaccination and was vaccinated |
| Other healthy | Healthy | Healthy animal presented for other reasons that do not fit in the vaccination or in the post-operative check categories |

**Supplementary material table 2**

Regular expression utilised to identify external laboratory diagnostic tests; insulin syringe sales, and refunds contained within product descriptions used by a network of UK companion animal-treating veterinary practices.

| Category | Regular expression |
| --- | --- |
| Diagnostic tests | \\sidex[x]?, ^idex[x]?, finn, nwl, tdds, ptds, ctds, capl, ^dvg\ss, ^fi\\s, ^gr\\s, ^ax\\s, ^lab\\s, ^lf\\s, ^cl\\s, [^a-z]ax\\s, axiom, abbey, ahvla, idx, in[ \|\\-]?hse, in[ \|\\-]?house, glasgow, bristol, pre[ \|\\-]?anaes, screen, request, accucheck, swab.*charcoal, charcoal.*swab, lab fee, suppression, profile, greenlab, phenobarb.*(monitor\|profile\|test\|level\|assay\|screen\|serum), test, ^labin[t]? , ^laboratory |
| Insulin syringes | caninsulin.*syr, syr.*caninsulin, caninsulin.*needle, needle.*caninsulin, vetpen.*needle, needle.*vetpen |
| Refunds | [^(non)]refund, incorrect, ^refund |

**Supplementary material table 3**

Frequency of prescription events for selected pharmaceutical families (PF) summarised to level of pharmaceutical class (PC) for dogs from a network of United Kingdom companion animal veterinary practices.

| Pharmaceutical  Family (PF) | Pharmaceutical  Class (PC) | Number of  PC prescription events (% of PF) |
| --- | --- | --- |
| Antibiotic | Clavulanic acid potentiated amoxicillin | 62475 (28.6) |
|  | Fusidic acid | 39712 (18.2) |
|  | Aminoglycoside | 26308 (12.0) |
|  | 1^st^ generation cephalosporin | 18305 (8.4) |
|  | Other antibiotic | 15685 (7.2) |
|  | Amoxicillin | 11631 (5.3) |
|  | Metronidazole | 10231 (4.7) |
|  | Clindamycin | 9978 (4.6) |
|  | Fluoroquinolone | 9430 (4.3) |
|  | Amphenicol | 4074 (1.9) |
|  | Potentiated sulphonamide | 3242 (1.5) |
|  | Tetracycline | 2494 (1.1) |
|  | 3^rd^ generation cephalosporin | 1943 (0.9) |
|  | Metronidazole - Spiramycin | 1653 (0.8) |
|  | Other beta-lactam | 774 (0.4) |
|  | Macrolide | 336 (0.2) |
|  | Lincosamide | 225 (0.1) |
|  | 2^nd^ generation cephalosporin | 91 (0.04) |
|  | Penicillin | 63 (0.03) |
|  | Clavulanic acid potentiated ticarcillin | 1 (0.0004) |
|  | Penicillin - streptomycin | 1 (0.0004) |
| Antimycotic | Imidazole | 21886 (59.5) |
|  | Polyene | 14342 (39.0) |
|  | Triazole | 443 (1.2) |
|  | Other antimycotic | 53 (0.1) |
|  | Allylamine | 31 (0.08) |
|  | Mitotic inhibitor | 8 (0.02) |
|  | Clotrimazole | 2 (0.01) |
|  | Fluconazole | 1 (0.003) |
| Ectoparasiticide | Neonicotinoid | 74651 (75.6) |
|  | Isoxazoline | 9306 (9.4) |
|  | Pyrethroid | 5237 (5.3) |
|  | Phenylpyrazole | 4364 (4.4) |
|  | Insect growth regulator | 3117 (3.2) |
|  | Spinosad | 1172 (1.2) |
|  | Oxadiazine | 657 (0.7) |
|  | Amitraz | 261 (0.3) |
|  | Semicarbazone | 3 (0.003) |
| Endectocide | Milbemycin | 110313 (96.9) |
|  | Avermectin | 3574 (3.1) |
| Endoparasiticide | Quinoline | 84525 (77.7) |
|  | Benzimidazole | 13823 (12.7) |
|  | Tetrahydropyrimidine | 10448 (9.6) |
|  | Octadepsipeptide | 6 (0.01) |

**Supplementary material table 4**

Frequency of prescription events for selected pharmaceutical families (PF) summarised to level of pharmaceutical class (PC) for cats from a network of United Kingdom companion animal veterinary practices.

| Pharmaceutical  Family (PF) | Pharmaceutical  Class (PC) | Number of  PC prescription events (% of PF) |
| --- | --- | --- |
| Antibiotic | 3^rd^ generation cephalosporin | 25696 (36.2) |
|  | Clavulanic acid potentiated amoxicillin | 15370 (21.6) |
|  | Amoxicillin | 8888 (12.5) |
|  | Fusidic acid | 7655 (10.8) |
|  | Aminoglycoside | 3175 (4.5) |
|  | Clindamycin | 2776 (3.9) |
|  | Fluoroquinolone | 2146 (3.0) |
|  | Other antibiotic | 1880 (2.6) |
|  | Metronidazole | 934 (1.3) |
|  | Amphenicol | 901 (1.3) |
|  | Tetracycline | 806 (1.1) |
|  | 1^st^ generation cephalosporin | 276 (0.4) |
|  | Metronidazole - spiramycin | 274 (0.4) |
|  | Lincosamide | 156 (0.2) |
|  | Other beta-lactam | 51 (0.1) |
|  | Macrolide | 38 (0.1) |
|  | Potentiated sulphonamide | 34 (0.1) |
|  | Penicillin | 21 (0.03) |
|  | 2^nd^ generation cephalosporin | 10 (0.01) |
|  | Rifamycin | 1 (0.001) |
| Antimycotic | Polyene | 1524 (58.4) |
|  | Imidazole | 1063 (40.7) |
|  | Triazole | 23 (0.9) |
|  | Allylamine | 1 (0.04) |
| Ectoparasiticide | Neonicotinoid | 36053 (64.5) |
|  | Insect growth regulator | 8777 (15.7) |
|  | Phenylpyrazole | 7564 (13.5) |
|  | Pyrethroid | 1339 (2.4) |
|  | Spinosad | 1162 (2.1) |
|  | Oxadiazine | 999 (1.8) |
|  | Isoxazoline | 1 (0.002) |
| Endectocide | Milbemycin | 54955 (86.7) |
|  | Avermectin | 8446 (13.3) |
| Endoparasiticide | Quinoline | 48835 (81.4) |
|  | Benzimidazole | 4186 (7.0) |
|  | Octadepsipeptide | 4179 (7.0) |
|  | Tetrahydropyrimidine | 2797 (4.7) |

**Supplementary material table 5**

Frequency of prescription events for selected pharmaceutical families (PF) summarised to level of pharmaceutical class (PC) for rabbits from a network of United Kingdom companion animal veterinary practices.

| Pharmaceutical  Family (PF) | Pharmaceutical  Class (PC) | Number of  PC prescription events (% of PF) |
| --- | --- | --- |
| Antibiotic | Fluoroquinolone | 2215 (49.4) |
|  | Fusidic acid | 815 (18.2) |
|  | Aminoglycoside | 447 (10.0) |
|  | Penicillin | 360 (8.0) |
|  | Potentiated sulphonamide | 173 (3.9) |
|  | Other antibiotic | 127 (2.8) |
|  | Tetracycline | 112 (2.5) |
|  | Amphenicol | 74 (1.7) |
|  | Amoxicillin | 66 (1.5) |
|  | 3^rd^ generation cephalosporin | 24 (0.5) |
|  | 1^st^ generation cephalosporin | 18 (0.4) |
|  | Macrolide | 18 (0.4) |
|  | Metronidazole | 10 (0.2) |
|  | Clindamycin | 9 (0.2) |
|  | Other beta-lactam | 5 (0.1) |
|  | Clavulanic acid potentiated amoxicillin | 5 (0.1) |
|  | 2^nd^ generation cephalosporin | 3 (0.1) |
| Antimycotic | Polyene | 61 (51.7) |
|  | Imidazole | 49 (41.5) |
|  | Other antimycotic | 5 (4.2) |
|  | Triazole | 3 (2.5) |
| Ectoparasiticide | Insect growth regulator | 168 (59.0) |
|  | Neonicotinoid | 82 (28.8) |
|  | Pyrethroid | 30 (10.5) |
|  | Phenylpyrazole | 4 (1.4) |
|  | Isoxazoline | 1 (0.4) |
| Endectocide | Avermectin | 753 (98.4) |
|  | Milbemycin | 12 (1.6) |
| Endoparasiticide | Benzimidazole | 547 (98.4) |
|  | Quinoline | 6 (1.1) |
|  | Triazinetrione | 2 (0.4) |
|  | Octadepsipeptide | 1 (0.2) |

**Supplementary material table 6**

Summary of the ten most common pharmaceutical co-prescriptions for dogs, cats and rabbits at pharmaceutical family (PF) level, summarized from a network of companion animal veterinary practices in the UK. Grey shading indicates co-prescription combination not in ten most frequently prescribed for that particular species.

| Pharmaceutical  Family (PF) 1 | Pharmaceutical  Family (PF) 2 | Percentage of total co-prescription events | | |
| --- | --- | --- | --- | --- |
|  |  | Dog | Cat | Rabbit |
| Antibiotic | Anti-inflammatory | 13.9 | 11.4 | 27.1 |
| Endectocide | Ectoparasiticide | 11.9 | 14.2 |  |
| Endoparasiticide | Endectocide | 10.9 | 14.3 |  |
| Vaccine | Endectocide | 9.0 | 11.8 |  |
| Vaccine | Endoparasiticide | 7.8 | 11.0 | 3.4 |
| Endoparasiticide | Ectoparasiticide | 7.3 | 9.4 |  |
| Vaccine | Ectoparasiticide | 7.0 | 8.5 |  |
| Antimycotic | Antibiotic | 5.1 |  | 2.4 |
| Antimycotic | Anti-inflammatory | 5.1 |  | 2.4 |
| Vaccine | Anti-inflammatory | 1.8 |  |  |
| Endectocide | Anti-inflammatory |  | 1.8 |  |
| Ectoparasiticide | Anti-inflammatory |  | 1.7 |  |
| Endectocide | Antibiotic |  | 1.3 |  |
| Gastrointestinal | Anti-inflammatory |  |  | 16.4 |
| Gastrointestinal | Antibiotic |  |  | 7.1 |
| Neurological | Anti-inflammatory |  |  | 5.9 |
| Neurological | Gastrointestinal |  |  | 4.1 |
| Neurological | Antibiotic |  |  | 4.0 |
| Endoparasiticide | Antibiotic |  |  | 3.0 |
